# Prevalence and Awareness of Obesity and Bariatric Surgeries in the Northern Borders Region, Saudi Arabia

**DOI:** 10.7759/cureus.50261

**Published:** 2023-12-10

**Authors:** Ryanh H Alanazi, Malik A Hussain, Rayan H Alanazi, Saja R Alanazi, Rouh Maskhur K Alanazi, Manal S Fawzy

**Affiliations:** 1 College of Medicine, Northern Border University, Arar, SAU; 2 Department of Surgery, Northern Border University, Arar, SAU; 3 Department of Biochemistry, Northern Border University, Arar, SAU; 4 Unit of Medical Research and Graduate Studies, Northern Border University, Arar, SAU

**Keywords:** weight-loss intervention, high bmi, overweight, awareness, obesity, bariatric surgery

## Abstract

Introduction

Obesity is a complex health issue affecting millions worldwide, characterized by excessive body fat accumulation, often leading to various health complications. Bariatric surgeries are effective interventions for severe obesity, assisting patients in attaining substantial weight reduction and enhancing their overall well-being. This study aimed to assess obesity patterns and bariatric surgery prevalence in the Northern Borders region of Saudi Arabia to increase community knowledge and awareness about obesity and bariatric surgery.

Methods

This cross-sectional study included 386 residents in the Northern Borders region, Saudi Arabia. The participants completed a previously validated self-administered electronic questionnaire, and the confidentiality of the collected data was ensured.

Results

Nearly 58.3% of the participants (31-40 years), with a predominance of females, had a body mass index (BMI) >30, and 33.7% had undergone bariatric surgery. Most participants (92.5%) were aware that obesity is associated with significant medical issues, 98.2% appreciated that there is a surgical method to reduce weight, and 58.8% indicated that the procedure was not safe. Additionally, the majority of the respondents (57.0%) were not sure about the complications of weight-loss surgeries, and only 28.0% knew that surgeries for obesity and their complications may lead to death. Significant associations were found between age, education level, and BMI concerning the knowledge of obesity/bariatric surgery (p=0.003, 0.001, 0.002), respectively. However, gender and work status did not show such associations (p> 0.05).

Conclusion

Our study highlighted a lack of knowledge among the community regarding the safety, potential complications, and survival outcomes associated with obesity and bariatric surgery that could be due to ignorance and reluctance to pursue bariatric surgery to overcome morbid obesity. Significantly, the study found a relationship between age, education level, BMI, and knowledge of obesity and bariatric surgery.

## Introduction

In the last few decades, obesity has become one of the major public health issues worldwide. It dramatically impacts adults and adolescents and could cause premature morbidity and mortality [1]. Furthermore, it is associated with multiple comorbid diseases, including diabetes, hypertension, and dyslipidemia [2]. As a result, all this leads to compromising life quality and reducing life expectancy [3].

The World Health Organization (WHO) estimates that the prevalence of overweight and obesity is 68.2% and 33.7%, respectively, in Saudi Arabia [4]. In the Arab region, 25-40% of children and 66-75% of adults were overweight or obese, as mentioned in 2014 studies [5]. The high prevalence of obesity in the Saudi Arabian population indicates inadequate or ineffective preventive measures are being used [6].

Individuals with severe obesity (body mass index (BMI) of 40 or higher) often gravitate towards the most effortless strategies for weight reduction, yet it is associated with high postoperative risks that should be highly concerning. Moreover, it is most likely a short-term solution if long-term lifestyle modifications are not applied as a part of treatment [7]. Therefore, for patients to explore its advantages, they need to be aware of how lifestyle modification is essential in bariatric surgery [7].

Obesity, like other chronic illnesses, persists over extended periods and necessitates ongoing close monitoring to reevaluate the success of therapies, including bariatric surgery. The latter is a surgical intervention technique, which was introduced to offer a more long-term weight loss treatment. Regardless of the type of bariatric surgery used to treat obesity, it has been shown to result in significant weight loss, but its long-term effectiveness needs to be assessed [8].

The appropriate management of morbid obesity may significantly impact the health of patients and the healthcare system. Morbid obesity can be effectively managed with bariatric surgery [9]. For all patients who undergo bariatric surgery, multidisciplinary preoperative evaluations by the surgery departments, anesthesia, endocrine, and psychiatry is a basic protocol [10].

Studies have shown weight loss of 20-40 kg up to 10 years after surgery [11]. The obesity-related comorbidities such as hypertension, type 2 diabetes, lipid abnormalities, and obstructive sleep apnea, often significantly improve or disappear entirely after surgery. Additionally, bariatric surgery has been demonstrated to produce a favorable psychosocial impact on a person's life [12].

Complications are classified as either early (within 30 days of the following surgery) or late (over 30 days) [13]. These include nausea, vomiting, abdominal pain, intestinal obstruction, diarrhea, and nutritional deficiencies. Until and unless the patient commits to maintaining and observing regular physical activity and a diet control plan, the postoperative effects of bariatric surgery will not support the expectations [14]. Most cases require a nutritious diet with enough hydration, minerals, multivitamins, and fewer fats and carbohydrates [14]. Bariatric surgery can improve the quality of life, but long-term consequences must be avoided through proper follow-up [15].

## Materials and methods

Study sample and setting

This cross-sectional, observational study was conducted in the Northern Borders region, Saudi Arabia, from May to October, 2023. A self-administered questionnaire was used for data collection. The study was approved by the Bioethics Committee at the Northern Border University, Arar, Saudi Arabia (reference number: HAP-09-A-043, dated May 3, 2023). To estimate the prevalence of bariatric surgery and awareness regarding bariatric surgery and obesity, a total of 384 participants were recruited.

Sample size and inclusion criteria

The sample size was calculated using Raosoft's sample size online calculator (Raosoft Inc., Seattle, Washington, United States), and 384 individuals were required to reach a 95% confidence interval with a 5% margin of error. Both genders older than 18 years and under the age of 65 living in the Northern Borders region, regardless of nationality were included in the study.

Data collection

Data were collected through an online self-administered questionnaire, adopted from Albogami et al. [16]. It was developed on Google Forms (Google LLC, Menlo Park, California, United States), which included four main sections concerning (i) demographic data, (ii) the prevalence of bariatric surgery, (iii) knowledge regarding bariatric surgeries, and (iv) obesity. The questionnaire was shared on various social media platforms randomly between May 21, 2023, and July 21, 2023.

Statistical analysis

Data analysis was done using IBM SPSS Statistics for Windows, Version 24.0 (Released 2016; Armonk, New York, United States). Prior to entering into the software, the data were coded. The application of descriptive statistics resulted in a frequency and percentage representation of the data with a significance at p-value = 0.05. A chi-square test was employed to analyze categorical variables and ascertain the relationship between the groups.

## Results

Sociodemographic characteristics

A total of 386 participants completed the questionnaire. Most participants (30.8%) belonged to the age group of 31-40 years. About 72.0% were females, with more than half (54.9%) having a bachelor's degree. Regarding work status, more than half of the participants (54.1%) were unemployed (Table 1).

**Table 1:** Sociodemographic characteristics of the participants (n = 386) Data are presented as frequencies (n) and proportion (%).

Sociodemographic characteristics	Category	Frequency and proportion, n (%)
Age	18-25 years	118 (30.6%)
	26-30 years	58 (15.0%)
	31-40 years	119 (30.8%)
	41-60 years	85 (22.0%)
	60 and above years	6 (1.6%)
Gender	Female	278 (72.0%)
	Male	108 (28.0%)
Education level	High school	93 (24.0%)
	Diploma	57 (14.8%)
	Bachelor's degree	212 (54.9%)
	Master's degree	12 (3.1%)
	Doctorate	6 (1.6%)
	Others	6 (1.6%)
Work status	Employed	177 (45.9%)
	Unemployed	209 (54.1%)

Prevalence of bariatric surgery and BMI

The prevalence of bariatric surgery and the BMI of the participants are depicted in Table 2. The findings revealed that more than half of the respondents (58.3%) had a BMI >30. Although the majority of the respondents were obese, only 33.7% of the respondents had undergone bariatric surgery.

**Table 2:** Prevalence of bariatric surgery and BMI of the participants Data are presented as frequencies (n) and proportion (%)

Prevalence of bariatric surgery	Categories	Frequency and proportion, n (%)
Have you done bariatric surgery?	Yes	130 (33.7%)
	No	256 (66.3%)
BMI	<=18	20 (5.2%)
	18.1-25	71 (18.4%)
	25.1-30	70 (18.1%)
	>30	225 (58.3%)

Knowledge of obesity and bariatric surgery

Table 3 depicts the knowledge of obesity and bariatric surgery among the participants. The findings revealed that the vast majority (90.1%) knew that obesity is a disease, with the majority 92.5% being aware that obesity or overweight could cause significant medical problems. The majority of the respondents (66.6%) correctly indicated that there was a high likelihood of getting health problems if obesity was not managed or treated. The vast majority (98.2%) of the participants knew of the existence of surgical methods to reduce weight, and 75.9% of them knew that weight-loss surgeries improve obesity-related complications, with more than half (58.8%) of them noting that the procedures were not safe. The majority of the respondents (57.0%) were not sure about the complication percentage of weight-loss surgeries. Only 28.0% of the respondents knew that surgeries for obesity and their complications may lead to death, with the majority of them not sure of the likelihood of death.

**Table 3:** Knowledge of obesity and bariatric surgery among the participants Data are presented in frequencies (n) and proportion (%).

Knowledge about obesity and bariatric surgery	Categories	Frequency and proportion, n (%)
Do you believe that obesity is a disease?	Yes	348 (90.1%)
	No	30 (7.8%)
	I don't know	8 (2.1%)
Do you think being obese or overweight can cause significant medical problems? (hypertension, diabetes mellitus, elevated blood lipids, heart disease)	Yes	357 (92.5%)
	no	21 (5.4%)
	I don't know	8 (2.1%)
What is the likelihood of getting health problems if obesity is not managed or treated?	Low	14 (3.6%)
	Moderate	115 (29.8%)
	High	257 (66.6%)
Do you know that there are surgical methods to reduce weight?	Yes	379 (98.2%)
	No	3 (0.8%)
	I don't know	4 (1.0%)
Do you think that weight-loss surgeries can improve obesity-related complications?	Yes	293 (75.9%)
	No	19 (4.9%)
	I don't know	74 (19.2%)
Do you think weight-loss surgeries are safe procedures?	Yes	99 (25.6%)
	No	227 (58.8%)
	I don't know	60 (15.5%)
What do you think is the complication percentage of weight-loss surgeries	Less than 5%	122 (31.6%)
	20%-30%	20 (5.2%)
	More than 40%	24 (6.2%)
	I don't know	220 (57.0%)
Do you think that the surgeries for obesity and their complications may lead to death?	Yes	108 (28.0%)
	No	121(31.3%)
	I don't know	157 (40.7%)
What do you think is the likelihood of death?	Low	165 (42.7%)
	Moderate	186 (48.2%)
	High	21 (5.4%)

Association between demographic variables and knowledge of obesity and bariatric surgery

Table 4 depicts the relationship of age, gender, level of education, occupation, and work status with knowledge of obesity and bariatric surgery. The results established a significant association between age and the knowledge of obesity and bariatric surgery (p = 0.003); there was a statistically significant association between education level and knowledge of obesity and bariatric surgery (p = 0.001), and BMI was also found to be statistically associated with knowledge of obesity and bariatric surgery (p = 0.002). There was no statistically significant association between gender or work status and the knowledge of obesity and bariatric surgery (p > 0.05).

**Table 4:** The association between demographic variables and knowledge of obesity and bariatric surgery Data are presented as percentages. A chi-square test was applied. Significance: p<0.05

Variables	Category	Knowledge of obesity and bariatric surgery		
		High (%)	Low (%)	p-value
Age	18-25 years	56.4	43.6	0.003
	26-30 years	57.9	42.1	
	31-40 years	57.5	42.5	
	41-60 years	57.3	42.7	
	60 and above years	58.2	41.8	
Gender	Female	43.1	56.9	0.145
	Male	56.4	43.6	
Education level	High school	56.6	43.4	0.001
	Diploma	57.3	42.7	
	Bachelor's degree	59.4	40.6	
	Master's degree	59.5	40.3	
	Doctorate	59.8	40.2	
	Others	56.5	43.5	
Work status	Employed	41.4	58.6	0.121
	Unemployed	44.	56.	
BMI	<=18	41.5	58.5	0.002
	18.1-25	43.1	56.9	
	25.1-30	57.3	42.7	
	>30	59.6	40.4	

## Discussion

The study aims to measure and assess the pattern of obesity and the prevalence of bariatric surgeries among residents of the Northern Borders region, Saudi Arabia, and also to increase community knowledge and awareness about obesity and bariatric surgery. The sample for the current study primarily consisted of adults aged 31-40 years, with a predominance of females; most of them had bachelor's degrees and were unemployed.

The study findings revealed that 58.3% of the participants were obese with a BMI >30. The study has established a high prevalent rate of overweight/obesity among the residents of the Northern Borders region, Saudi Arabia. The findings are consistent with those of a study conducted in Saudi Arabia by Syed et al. that reported high prevalence rates of obesity attributed to unhealthy dietary habits/lifestyles and poor awareness [17]. This necessitates implementing educational and awareness programs at education entities and communities. 

The current study found that only 33.7% of the participants had undergone bariatric surgery, despite being aware of its use as a treatment option for weight reduction. This discrepancy might suggest that while many participants are informed about bariatric surgery, the understanding of its benefits, procedures, and outcomes may be low, particularly considering the majority of the participants were obese. The findings of this study are consistent with those of Alqahtani et al., which reported poor knowledge of bariatric surgery, in which 22.7% of the study subjects were unaware of the bariatric surgery to treat obesity in eastern Saudi Arabia [18].

Although the findings of the present study revealed that the majority of the respondents knew that obesity is a disease that could cause significant medical problems (92.5%) and that there was a surgical method to reduce weight (98.2%), more than half of the respondents (58.8%) indicated that the procedure was not safe. Additionally, the majority of the respondents (57.0%) did not know about the complication percentage of weight-loss surgeries. Only 28% of the respondents knew that surgeries for obesity and their complications may lead to death, with a majority of them not sure of the likelihood of death. The study's findings point out poor knowledge about obesity and bariatric surgery, mainly information related to their safety, complications, and the survival chances and outcomes of the procedures. The findings are consistent with those of BaMehriz et al., who found that although bariatric surgery is a safe/effective procedure for weight loss among those with morbid obesity, experience, and facilities may significantly decrease the subsequently expected complications [19]. However, the overall safety, practicability, and outcomes are still unknown for the Saudi experience. Also, this finding is consistent with others who found a significant gap in understanding and awareness regarding bariatric surgery among both medical professionals and the general population [20-22].

The sociodemographic variables of age, level of education, and BMI were significantly associated with knowledge of obesity and bariatric surgery. The study established a significant association between age, education level, and BMI with knowledge of obesity and bariatric surgery (p = 0.003, 0.001, and 0.002, respectively). However, there was no significant association between gender and work status with knowledge of obesity and bariatric surgery.

The poor level of knowledge regarding obesity and bariatric surgery points to the ignorance among the community and the reluctance to pursue bariatric surgery for morbid obesity. The study finds the level of education and the community perception imperative in promoting knowledge and awareness about obesity and bariatric surgery through multidisciplinary intervention, including public awareness, healthcare givers, and health educators. Our findings and conclusions have potential alignment with other studies conducted in different areas of Saudi Arabia, as illustrated in Table 5.

**Table 5:** Summary of studies in Saudi Arabia concerning assessment of awareness of obesity and bariatric surgery

Authors, year	Study location	Sample size	Data collection	Reported outcome(s)
Mahmoud et al., 2021 [7]	Different regions of Saudi Arabia	713	A structured, pre-coded, closed-ended, pilot-tested online questionnaire	- More than half of the affected individuals choose elective bariatric surgeries as their preferred solution for obesity. However, the understanding and attitude of the Saudi population towards bariatric surgeries - in terms of recognizing their benefits, potential complications, indications, and various procedures - remains less than ideal.
Albogami, 2021 [16]	Riyadh city	512	A self-administrated electronic questionnaire (Google survey)	- The study population demonstrated satisfactory awareness of obesity and its associated health complications. However, the understanding of bariatric surgery is inadequate. Dissemination of “evidence-based information” concerning the safety of bariatric surgery through several platforms like TV broadcasts, social media apps, and various awareness campaigns is highly recommended.
Abouhamda, 2016 [23]	Jeddah city	474	Online questionnaires	- A significant portion (74%) of those surveyed demonstrate limited understanding of bariatric surgery, while just over half (50.8%) thoroughly comprehend obesity prevention measures.
Alqurashi, 2017 [24]	Throughout Saudi Arabia	790	Online questionnaires	- Subject awareness regarding obesity and proactive prevention is substantial, but there is a noticeable lack of positive understanding toward bariatric surgery. This indicates there is a need for developing effective educational initiatives about obesity and Bariatric surgeries to manage obesity and its related complications, as well as enhance the accessibility of weight loss surgical procedures.
Aldawqi, 2018 [25]	Riyadh city	500	Self-administered questionnaire was distributed to adult citizens of Riyadh city	- Most surveyed tended to exaggerate the dangers associated with bariatric surgery and didn't view it as the optimal weight loss solution. It is advisable to implement health education initiatives to rectify these misbeliefs and assure people about its comparative safety and anticipated health advantages.
Alfadhel, 2020 [26]	Different regions of Saudi Arabia	891	Online questionnaires	- In Saudi Arabia, awareness about obesity is generally high, and the majority of Saudis prefer adopting a balanced diet instead of resorting to surgical procedures. More people fall within the normal body mass index (BMI) range of 18.5-25 in the present study.
Alrashid, 2021 [27]	Tabuk region	274	An electronic self-administered questionnaire	- Obesity/overweight health knowledge is poor among obese and those who have experienced bariatric surgeries in Saudi Arabia, which imposes further efforts at the community level. - Knowledge of bariatric surgery-associated factors, including health benefits and complications, is low in the Tabuk area, necessitating educational interventions.
Alenezi, 2022 [28]	Northern areas of Saudi Arabia: Hail, Aljawf, Tabuk, and Arar.	280	Self-administered survey form (Google form) on the electronic devices of the research team at the participant’s workplace	- Study results indicate that a lack of understanding of bariatric surgery contributes to various worries and obstructions to referrals among doctors. As such, it is advised that the background knowledge of primary care physicians be enriched via continuous medical education, seminars, and other appropriate platforms, with particular emphasis on obesity care in the course content. - It's necessary to conduct an integrated survey across different provinces of Saudi Arabia to tailor specific training needs that align with each region's conditions.
Alwhibi, 2022 [29]	Riyadh city	223	Self-filled questionnaire distributed to family medicine physicians from cluster two healthcare facilities	- Family medicine doctors based in Riyadh's second cluster show a diverse range of knowledge and favorable opinions concerning bariatric surgery. The study suggests enhancing these physicians' understanding and cognizance of the advantages, outcomes, methods, and reasoning behind bariatric surgery. This can be achieved by involving them in fundamental and advanced health education workshops, and courses focused on the surgical treatment of obesity and metabolic disorders.
Almontashri, 2023 [30]	Makkah Almukarramah	388	An online questionnaire via Google Forms	- The participants showcased a strong understanding of obesity and its impact on health. They also displayed good knowledge about bariatric surgery as a successful method for weight loss, though they showed a preference for non-surgical approaches. This implies their consideration of the potential complications of bariatric surgery. Nevertheless, the findings revealed a shortfall in the awareness of indications following surgery and the necessary lifestyle alterations. This underscores the importance of all-inclusive patient education and guidance.
Jastaniah, 2023 [31]	Western region of Saudi Arabia	1122	Online questionnaire	- The collective understanding within the general population of the Western region of Saudi Arabia concerning obesity and complications linked to bariatric surgery was deemed adequate. Nevertheless, further research must be carried out across Saudi Arabia.

Although the present study has provided valuable updates on obesity and bariatric surgery in the Northern Borders region, it has some limitations. Firstly, its cross-sectional design can only establish the relations between factors but not causalities. Secondly, since the study utilized online self-administered questionnaires to collect data, it was challenging to discover insights or observe the behavior of respondents in their natural settings. Also, the study findings cannot be generalized to the entire Saudi Arabian population, considering that it was conducted in only one region.

## Conclusions

The present study found that 58.3% of the participants were obese with a BMI >30, and the prevalence of bariatric surgery was 33.7% in the Northern Borders region of Saudi Arabia. The study points out the existence of poor knowledge about obesity and bariatric surgery, mainly information related to their safety, complications, and the survival chances and outcomes of the procedures, which can be attributed to the ignorance among the community and the reluctance to pursue bariatric surgery for morbid obesity. The study established a significant association of age, education level, and BMI with knowledge of obesity and bariatric surgery. Providing comprehensive education and empowering both physicians and patients with enhanced knowledge is expected to have a profound effect on patients' perspectives and safety apprehensions related to this type of surgical procedure.
